# Controlling Droplet Evaporation in Aerosol Jet Printing to Understand and Mitigate Overspray

**DOI:** 10.1002/smsc.202500069

**Published:** 2025-03-18

**Authors:** Bella I. Guyll, Brayden L. Sanford, Cary L. Pint, Ethan B. Secor

**Affiliations:** ^1^ Department of Mechanical Engineering Iowa State University Ames Iowa 50011 USA

**Keywords:** additive manufacturing, microfabrication, multiphase flow, nanomaterial inks, printed electronics

## Abstract

Aerosol jet printing is an additive manufacturing technique with broad materials compatibility, high resolution, and complex geometric capabilities. Despite these advantages, even optimized prints are susceptible to overspray, in which sparse deposition of material outside the primary pattern limits precision and quality for high‐value applications. Herein, a method is presented to overcome this by loading the sheath gas with solvent vapor before entering the printhead. This reduces droplet drying in the aerosol phase at the periphery of the aerosol stream, improving line edge morphology, pitch, porosity, and surface finish. This is demonstrated to reduce the overspray extent for a water‐based polyimide ink by up to 70 ± 2.3% and decrease the resistivity of a solvent‐based silver ink by 34 ± 13%. The ability to regulate droplet evaporation in flight offers versatile control, facilitating a wider range of process parameters and ink chemistries. These experimental results are backed by theoretical analysis and numerical modeling, providing a more refined and generalizable understanding of the underlying process physics. This enables tailored outcomes for a range of challenges including high aspect ratio and high‐density patterning with improved surface finish and material functionality for compelling applications in printed and hybrid electronics.

## Graphical abstract

Saturating the sheath gas during aerosol jet printing limits aerosol‐phase evaporation, reducing overspray and improving overall print quality. This experimentally validates nonuniform sheath‐induced drying as a key mechanism during printing, with implications for high‐resolution patterning, electrical conductivity, and surface finish. This combination of experiments, theory, and numerical modeling provides a toolset to improve diverse and complex printing applications.

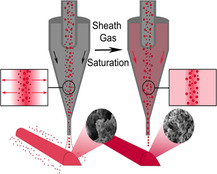

## 1 Introduction

Digital printing describes a rapidly expanding suite of capabilities, enabling the versatile production of electronic devices to support continued advancements in the automotive, energy, healthcare, communications, and aerospace industries. Aerosol jet printing (AJP) is a direct‐write, noncontact, digital printing technology that supports complex fabrication requirements for these sectors including precise, multimaterial, high aspect ratio, and conformal devices.^[^ ^1^, ^2^, ^3^, ^4^, ^5^, ^6^ ^]^ AJP uses functional liquid‐phase inks to produce micron‐sized droplets (1–5 μm) by ultrasonic or pneumatic atomization.^[^ ^7^ ^]^ These droplets are then transported on a carrier gas flow to the printhead, where they are focused using an annular sheath gas. The sheath gas serves to concentrate and accelerate the aerosol droplets while preventing impingement on the interior of a converging nozzle. Upon exiting the 100–300 μm nozzle, the jet containing aerosol droplets impinges on a surface 1–5 mm away, where the droplets coalesce into a printed feature.^[^ ^8^, ^9^ ^]^ This supports rapid iteration of designs and parameters, high‐resolution (6–100 μm), broad materials compatibility, and conformal deposition capabilities, offering benefits compared to traditional manufacturing methods.^[^ ^10^, ^11^, ^12^, ^13^ ^]^ These have been leveraged for a wide range of functional electronics, including diverse sensing, energy, and high frequency devices.^[^ ^14^, ^15^, ^16^, ^17^, ^18^, ^19^, ^20^, ^21^, ^22^ ^]^ Despite these advantages, a limited understanding of AJP's underlying physics continues to challenge process control and customization, especially for demanding and high‐value applications.^[^ ^23^, ^24^ ^]^

One mechanism in AJP that remains poorly understood is droplet evaporation within the printhead. This phenomenon plays a key role in process outcomes by contributing to the stable printing window, ink formulation, and tradeoffs between deposition rate and resolution.^[^ ^25^ ^]^ Without this fundamental awareness, intensive efforts to optimize resolution have often led to low material throughput, high variability, and excessive overspray, preventing multifaceted optimization and limiting translation to manufacturing environments.^[^ ^26^, ^27^ ^]^ Thus, understanding this mechanism is crucial for a theory‐driven approach to optimize complex interactions between ink formulation, printing parameters, and machine design.^[^ ^28^ ^]^

It is empirically known that solvent volatility plays a critical role in AJP, and a better fundamental understanding is a prerequisite to facilitate translation from research settings to manufacturing environments.^[^ ^29^ ^]^ Recent studies have highlighted the importance of aerosol‐phase evaporation on AJP outcomes. Our previous work demonstrated the effect of varying the printhead temperature to allow higher deposition rates without compromising resolution.^[^ ^29^, ^30^ ^]^ This changed the solvent vapor pressure and was effective in shifting the bulk state of printing away from a “wet” condition. Tailoring evaporation is a compelling method to affect print properties because the micron‐sized droplets can dry rapidly during flight. This results in the decoupling of the physicochemical properties of the original ink in the cartridge from those corresponding to the deposited material.^[^ ^8^ ^]^ Current practices to optimize the printing process, which inherently require tailoring droplet drying, include amending ink volatility, adjusting print parameters, and changing the volume fraction of aerosol. However, these techniques require substantial empirical efforts and often necessitate printing in dry conditions, leading to low deposition rates and prolonged production times while often exacerbating overspray. Thus, more sophisticated methods to modulate evaporation, particularly ones responsive to the spatial dependence of aerosol drying, are sought to improve understanding and control of the AJP process.

One possible method to achieve this is by lessening the amount of solvent evaporation required to saturate the vapor pressure within the printhead. Previous work has demonstrated that evaporation has a significant effect on printing characteristics by altering solvent chemistry and solid volume fraction, and thus rheology.^[^ ^31^ ^]^ These long‐term temporal effects can be reduced, and reliability improved, by reintroducing solvent through the carrier gas flow. While this alteration demonstrates the significance of evaporation in AJP, it neglects to address and understand the significant droplet evaporation that occurs downstream in the process, due to the introduction of the sheath gas within the printhead. Solvent bubblers are often implemented on the aerosol gas line to mitigate evaporation and process drift demonstrating the ease of bubbler integration with AJP.^[^ ^32^ ^]^ A proof of concept experiment using a solvent bubbler on the sheath gas line suggested that droplet evaporation is meaningfully affected by the presence of vapor in the gas flow; however, this was not further investigated to understand droplet drying within the printhead or how it can be exploited to modulate print quality, particularly overspray and rheology.^[^ ^30^, ^33^ ^]^

Here, we implement a sheath gas bubbler to refine our fundamental understanding of spatial drying variation in AJP and its links to print morphology, particularly overspray. The selected bubbler solvent is consistent with the ink chemistry and thus serves to reduce droplet evaporation at the periphery of the aerosol flow. The results demonstrate that saturating the sheath gas significantly reduces the prevalence of overspray, a finding supported and explained by an associated theoretical analysis. A numerical model to capture these effects indicates that drying occurs predominantly on the outer droplets, and the accompanying decrease in Stokes number leads to droplet divergence from the jet axis during impaction, resulting in overspray. Sheath gas saturation is shown to be effective for a variety of ink chemistries and offers additional benefits such as improved droplet impaction efficiency, microstructure, and surface finish. This methodology supports the use of lower carrier gas flow rates and higher focusing ratios without adverse effects attributed to excessive drying. A theory‐based approach to tailoring droplet characteristics could be integrated with other process amendments, such as acoustic focusing and standard optimization principles, to further improve overspray and morphology.^[^ ^13^, ^34^ ^]^ These basic process improvements are extended to electronics fabrication, with improved electrical conductivity and higher density patterning possible via sheath gas saturation. Altogether, this work advances our understanding of AJP and provides practical and effective experimental tools to better engineer physical mechanisms within the printhead to improve a variety of morphological and functional print outcomes.

## 2 Results and Discussion

### 2.1 Linking Droplet Evaporation to Overspray via Sheath Gas Saturation

**Figure 1:**
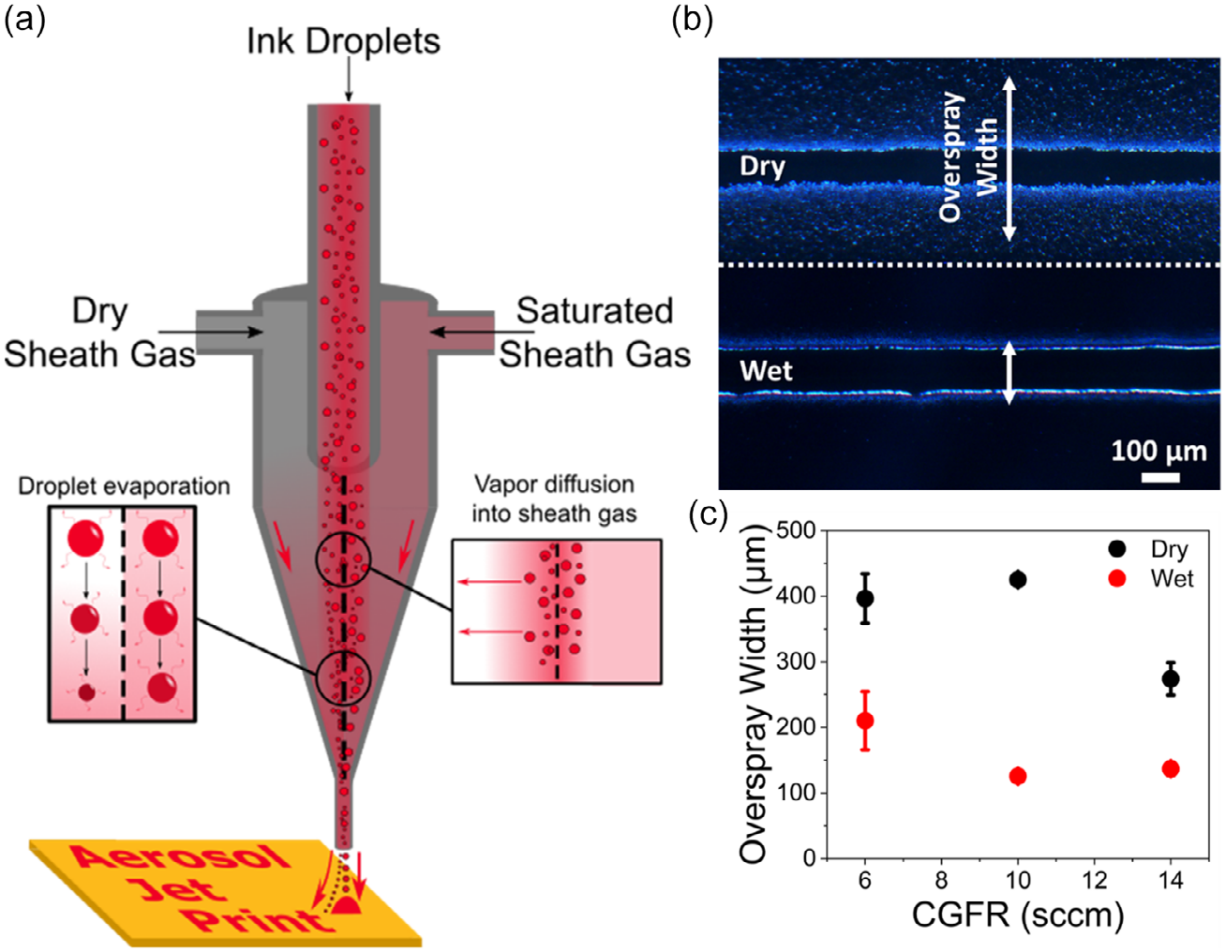
a) Schematic depicting AJP setup and processes with a dry (left) and saturated (right) sheath gas. b) Microscope images under normal conditions (top) and saturated conditions (bottom) (CGFR: 10 sccm, FR: 5, print speed: 1 mm s^−1^). c) Overspray width versus CGFR of PI ink (FR: 5, print speed: 2 mm s^−1^).

In this study, a solvent bubbler (Figure S1, Supporting Information) is used to partially saturate the sheath gas with solvent vapor prior to entering the printhead. The amount of material entering the printhead is held relatively constant by implementing an optics cell in‐line and maintaining the optical scattering readings for each condition.^[^ ^35^ ^]^ The effect of sheath gas saturation on downstream process physics is shown schematically in **Figure** **1**a. Presaturating the sheath gas reduces the amount of solvent vapor required from the aerosol source to reach the saturation vapor pressure of the system, providing a relatively straightforward method to tailor aerosol drying in flight prior to impaction.

An aqueous polyimide (PI) ink was chosen for the initial validation of sheath gas saturation to highlight overspray occurrence, reduce porosity effects on deposition rate measurements, and support the expansion of ink development and optimization of water‐based formulations. During printing, the carrier gas flow rate (CGFR) controls the flow of aerosol droplets into the printhead, while the sheath gas flow rate (SGFR) controls an annular shielding gas that constricts the aerosol stream and prevents droplet impaction on internal surfaces. At typical operating conditions, with a CGFR of 10 standard cubic centimeters per minute (sccm) and focusing ratio (FR, SGFR/CGFR) of 5, a single trace printed at a speed of 1 mm s^−1^ exhibits high prevalence of overspray, indicated by the bright colored specks surrounding the printed feature in dark field microscope images (Figure 1b, top). When the sheath gas is passed through a bubbler upstream of the printhead, which will be referred to as the saturated or “wet” condition, the majority of the functional material ends up as part of the printed feature, with dramatically reduced overspray (Figure 1b, bottom). This contrast provides evidence that, in the control, or “dry,” condition, droplets on the periphery of the carrier flow dry out when in contact with the vapor‐depleted sheath gas. The reduction in droplet size accompanying rapid evaporation reduces the droplet inertia. If the droplet size falls below the critical Stokes number, this can result in a failure to impact or cause deviation from the jet axis leading to overspray or rebounded particles.^[^ ^11^ ^]^ In comparison, sheath gas saturation successfully disrupts the most egregious droplet drying, allowing for a 70 ± 2.3% reduction in the lateral extent of the deposited pattern in a representative case (CGFR: 10 sccm, print speed: 2 mm s^−1^), described here as the overspray width (Figure 1b,c). Here, data points represent the average of three distinct samples while error bars represent the standard deviation between them as outlined in Section 4. The discrepancy between the two conditions is expected to be particularly prominent when operating in dry conditions, such as high FR, low CGFR, high vapor pressure solvents, and low volume fraction of aerosol.^[^ ^29^ ^]^

**Figure 2:**
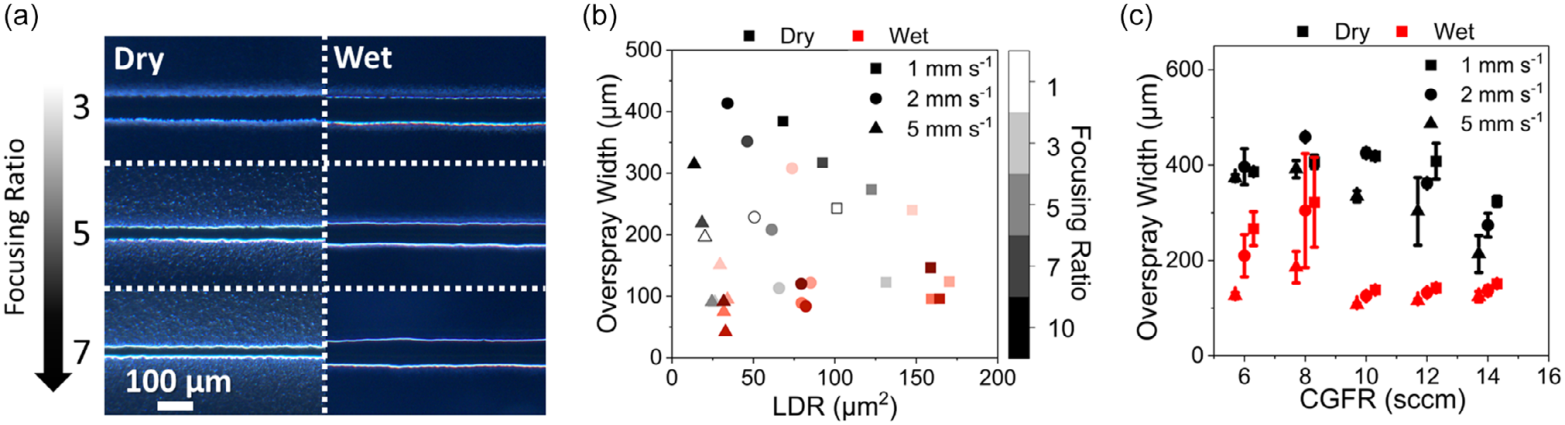
a) Microscope images of PI ink in dry (left) and wet (right) conditions at varying focusing ratios (CGFR: 10 sccm, print speed: 2 mm s^−1^). b) Overspray width versus linear deposition rate (LDR) (CGFR: 10 sccm). c) Overspray width versus CGFR (FR: 5).

Incidentally, increasing the FR and reducing the CGFR are both common practices to achieve better resolution empirically without a full appreciation of their consequences. This effect is evidenced by the microscope images in **Figure** **2**a (left) that detail how sheath gas flow influences print quality. As the FR is increased, the main feature size gets smaller, but the overall footprint and quality of the line diminish drastically. This could potentially lead to subpar printing outcomes, such as electrical shorting and high porosity. This is particularly apparent when compared to the wet condition in Figure 2a (right), in which higher focusing ratios reduce the overspray footprint of the printed trace with only a minor effect on the main feature width.

Saturating the sheath gas enables printing in otherwise dry conditions. This allows for the implementation of higher FRs, leading to higher resolution prints, without the adverse effects of excessive drying. Figure 2b demonstrates that, in the wet condition, higher focusing ratios allow higher speed‐normalized deposition rates, or linear deposition rates (LDR), to be used without a commensurate increase in the footprint through the reduction of overspray and nonimpacting particles. This trend holds for other standard operator controls to improve resolution, such as curtailing CGFRs. Figure 2c illustrates that saturating the sheath gas plays a significant role in reducing droplet drying at the lower end of CGFRs. This contrast becomes less significant as CGFR is increased, for these conditions beginning at 10 sccm. These results support the theory that droplet drying occurs primarily at the periphery of the carrier gas flow due to rapid evaporation relative to the diffusion timescale. Higher errors are seen at the lowest flow rates, as overspray width is linked more closely with where the droplets land or if they fail to impinge altogether. At the higher flow rates, larger standard deviations are indicative of liquid phase spreading where excess solvent causes the deposited material to flow over the substrate, resulting in an irregular line edge morphology. In flight tailoring of evaporation characteristics is thus an efficacious method for tuning the aerosol characteristics and print behavior. This corroborates previous theoretical treatments of droplet drying and abates some of the most egregious effects of overdrying, while providing a pathway to streamline the use of coarsely optimized ink formulations. In this case, sheath gas saturation supports high‐quality AJP from an ink containing only water as a solvent, which is not otherwise straightforward due to volatility effects, and offers clear benefits for cost, safety, and environmental sustainability.

### 2.2 Theoretical and Numerical Analysis of Droplet Evaporation in Confined Flows

#### 2.2.1 Nondimensional Analysis

The experimental results validate the conceptual understanding of overspray and the influence of sheath gas saturation described earlier. To align these empirical results with a better theoretical grounding, a more formal analysis of droplet evaporation within the printhead is developed here. This analysis considers laminar flow of an aerosol through a cylinder to support an intuitive and simple understanding of the process. The concentration of solvent vapor is governed by the reaction‐advection‐diffusion equation, with a volumetric source term representing evaporation from aerosol droplets.

(1) $$\frac{\partial c}{\partial t}+\nabla \times \left(uc\right)=\nabla \times \left(D\nabla c\right)+Q_{m}$$

(2) $$Q_{m}=4\pi aD\varphi_{n}\left(c_{s}-c\right)$$

in which *c* is the solvent vapor concentration, u the velocity, *D* the diffusivity, and $Q_{m}$ the volumetric source, which is based on the droplet size (*a*), droplet number density ($\varphi_{n}$), and solvent vapor saturation concentration ($c_{s}$).

Nondimensionalizing the reaction‐advection‐diffusion equation yields

(3) $$\frac{1}{{\text{Fo}}_{m}}\frac{\partial \tilde{c}}{\partial \tilde{t}}=\tilde{\nabla }\times \left(\tilde{\nabla }\tilde{c}\right)-{\text{Pe}}_{m}\tilde{\nabla }\times \left(\tilde{u}\tilde{c}\right)+\frac{1}{{\text{Fo}}_{m}}{\tilde{Q}}_{m}$$

in which the $\tilde{c}$ notation denotes a nondimensional value, in this case nondimensional concentration. For this, we nondimensionalize concentration with respect to the saturation value ($c=c_{s}\tilde{c}$), position based on the radial coordinate ($x=R\tilde{x}$), and time with respect to the evaporation timescale $\left(t=\tau_{e}\tilde{t}\right)$. Considering Poiseuille flow, the velocity is in the axial direction, and the spatial derivatives can be separated as
(4)

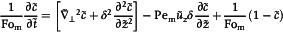




In which ${\text{Fo}}_{m}$ is the mass Fourier number, *δ* is the geometrical aspect ratio (R/L), ${\text{Pe}}_{m}$ is the mass Peclet number, and ${\tilde{u}}_{z}$ is nondimensionalized based on the average velocity, such that ${\tilde{u}}_{z}=2\left(1-{\tilde{r}}^{2}\right)$. More detail for these characteristic dimensionless numbers is given in **Table** **1**.

**Table 1:** Dimensionless numbers, their equations, and applicability to AJP droplet drying physics.

Dimensionless number	Equation	Description
Fourier number (mass)	${\text{Fo}}_{m}=\frac{\tau_{e}D}{R^{2}}$	Evaporative to diffusive timescale
Aspect ratio	$\delta =\frac{R}{L}$	Geometric aspect ratio
Peclet number (mass)	${\text{Pe}}_{m}=\frac{RU}{D}$	Diffusive to advective mass transport
Advection term	${\text{Pe}}_{m}\delta$	Diffusive to advective timescale
Evaporation timescale	$\tau_{e}=\frac{1}{4\pi aD\varphi_{n}}$	–

Under the assumption of a small aspect ratio ($\delta^{2}\ll 1$) and steady‐state conditions, rearranging the equation yields
(5)

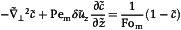




Intuitively, the dimensionless value modifying the advection term simply describes the diffusive to advective timescale, and the dimensionless value modifying the source term describes the diffusive to evaporative timescale. Three mechanisms drive the evolution of this system—the undersaturation of the solvent vapor ($1-\tilde{c}$), the Fourier number, and the product of the Peclet number and aspect ratio.

If solvent can evaporate from droplets (undersaturation), how the system evolves depends on the Fourier number, Peclet number, and aspect ratio. To represent fairly typical values for AJP, we consider a droplet radius of 1 μm, solvent vapor diffusivity of 7 × 10^−6^ m^2^ s^−1^, and aerosol volume fraction of 5×10^−5^.^[^ ^36^ ^]^ We consider two geometries in **Table** **2**, denoted as wide and narrow (*R* = 3 and 0.5 mm, respectively), and two total flow rates (*f*_t_ = 90 and 15 sccm).

**Table 2:** Analysis of reaction‐advection‐diffusion during AJP under different geometric and flow assumptions.

Variable	Condition 1	Condition 2	Condition 3	Condition 4
R [mm]	3	3	0.5	0.5
L [mm]	20	20	20	20
$f_{t}\;\left[\text{sccm}\right]$	90	15	90	15
$U_{\text{avg}}\;\left[m\;s^{‐1}\right]$	0.119	0.020	1.910	0.318
$1/{\text{Fo}}_{m}$	1350	1350	37.5	37.5
${\text{Pe}}_{m}\delta$	3.410	0.568	3.410	0.568
$1/{\text{Fo}}_{m}{\text{Pe}}_{m}\delta$	396	2375	11	66

In Equation (5), the dimensionless coefficient modifying the evaporation source term is much greater than those corresponding to advection and diffusion, implying that the evaporative term dominates under these typical conditions for AJP, or more explicitly, that droplet drying happens very quickly when the solvent vapor concentration is below saturation. Conditions that exacerbate this disparity in timescales include wider flow chambers and lower flow rates, while the narrower flow chamber and higher flow rates get these mechanisms closer to parity (≈10^1^–10^2^) by decreasing diffusion and process timescales, respectively.

In the AJP printhead, there is radially stratified flow with the inner carrier gas surrounded by an outer sheath gas. A high value for the inverse Fourier number implies that evaporation is localized near the periphery of the aerosol stream, due to slow diffusion of vapor outward from the center. A low value for the product of Peclet number and aspect ratio indicates a longer residence time within the printhead. Without changing the aspect ratio, or significantly altering the droplet size and number density, it is difficult to avoid significant drying at the outside of the aerosol stream. As discussed in our previous work, these dry droplets exhibit lower Stokes number, resulting in greater deviation in their trajectories from the jet axis.^[^ ^29^ ^]^ Moreover, complete drying would influence contact mechanics, limiting the amount of kinetic energy that can be dissipated via deformation and increasing the likelihood that dried particles rebound off the surface. Overspray is the manifestation of these effects during printing.

#### 2.2.2 Numerical Modeling

This conceptual understanding is borne out by a simplified numerical model of the printhead. We model the printhead as coaxial laminar flow containing an inner aerosol stream and an outer, initially dry, sheath gas. For the aerosol stream, we consider an “ink” composed of a single solvent, with ideal gas behavior for the solvent vapor, and we consider evaporation of a monodisperse population of droplets based on the local far‐field vapor concentration in the gas flow. We assume a solids volume fraction of 0.02 to distinguish between dry droplets and numerical diffusion, and use physical chemistry properties of xylenes for the solvent. This model is implemented in COMSOL Multiphysics.

**Figure 3:**
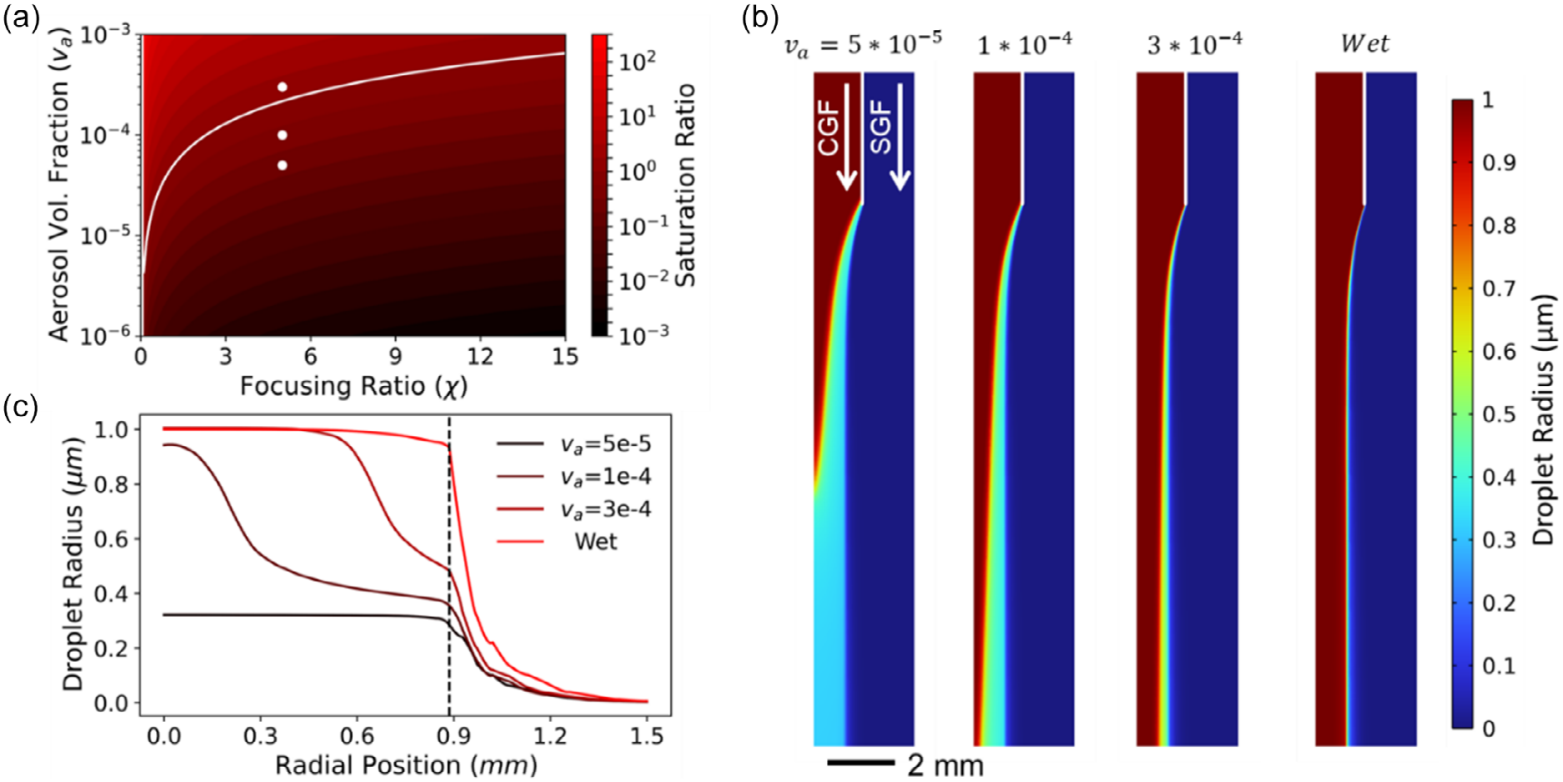
Theoretical analysis of droplet evaporation. a) Saturation ratio plotted as a function of aerosol volume fraction and focusing ratio for xylenes, showing the driving force for bulk evaporation. b) Plots of the droplet size as a function of position for numerical simulations. The interior CGF contains droplets, and after joining the annular SGF, these droplets begin evaporating. Simulation results are shown for the three standard conditions at different aerosol volume fractions (shown in (a)), along with the “wet” condition corresponding to a saturated sheath gas. c) Droplet size at the chamber exit, plotted against radial position. The dashed line indicates the edge of the droplet flow, with nonzero values outside this region arising from numerical diffusion.

The first parameter to keep in mind is the saturation ratio, defined in our prior work as the ratio of the amount of solvent present in liquid form to the amount of solvent required to saturate the vapor.^[^ ^29^ ^]^ For a single‐component solvent, this gives

(6) $$\text{Saturation ratio}=\frac{\upsilon_{a}}{c_{s}V_{m}\chi }$$

in which $v_{a}$ is the aerosol volume fraction (related to the number density of droplets), $V_{m}$ the molar volume, and *χ* the focusing ratio. This describes the overall drive toward evaporation, while the previously discussed analysis of the reaction‐advection‐diffusion equation captures the spatial and temporal influences. Printing often occurs in a condition of light undersaturation and for a given solvent depends on both the aerosol volume fraction and focusing ratio. The theoretical saturation ratio for xylenes is shown in **Figure** **3**a, with the line corresponding to a value of unity. The points in Figure 3a correspond to aerosol volume fractions of 5 × 10^−5^, 1 × 10^−4^, and 3 × 10^−4^, representing deep undersaturation, moderate undersaturation, and oversaturation.

These three conditions were modeled, with the resulting droplet size shown in Figure 3b. In the first case, droplets dry completely prior to exiting the flow chamber; in the intermediate case, droplets around the outside of the aerosol stream are nearly completely dried, while the central droplets remain near their original size; in the oversaturated case, there notably remain droplets around the periphery that experience some drying, with very little size change for the inner droplets, highlighting the fact that some droplets exhibit significant drying even when the bulk condition has excess solvent due to spatial disparities. When the sheath gas is saturated with solvent vapor, evaporation is completely shut down because there is no solvent vapor gradient that allows diffusion and local deviation from vapor‐liquid equilibrium. The droplet size at the chamber exit for each case is shown in Figure 3c, highlighting the strong effect of sheath gas saturation in promoting a more uniform droplet size distribution and, in particular, reducing droplet evaporation at the periphery of the aerosol stream that leads to overspray.

These model results, and corresponding intuition, align with the experimental observations. At low CGFR, a reduced aerosol volume fraction and high residence time within the printhead lead to significant drying. Lower total flow rates also exacerbate impaction‐based spreading, with a low jet velocity reducing the droplet Stokes number and leading to droplet impingement further from the jet axis. At higher FR, the saturation ratio is reduced, but the process timescale is also reduced, which have counteracting effects. More drying at high FR can lead to completely dried droplets at the periphery of the aerosol stream. In cases where these rebound from the substrate, this can lead one to conclude that the resolution is improved, even though this condition is clearly problematic from a material usage, environmental, and safety viewpoint. As it pertains to material functionality, near‐dry deposition can lead to a more granular morphology and could cause higher roughness in printed patterns. While saturating the sheath gas is not the optimal solution for all conditions—as exemplified by our previous work with a heated printhead, in which pushing droplet evaporation in the opposite direction yielded improvements in printing via reduced liquid‐phase spreading—this does provide a valuable tool to better control the printing process and further understanding of the underlying mechanisms that influence process optimization, printer design, and ink formulation.^[^ ^29^ ^]^

### 2.3 Generalizing Evaporation Control to Disparate Ink Chemistries

**Figure 4:**
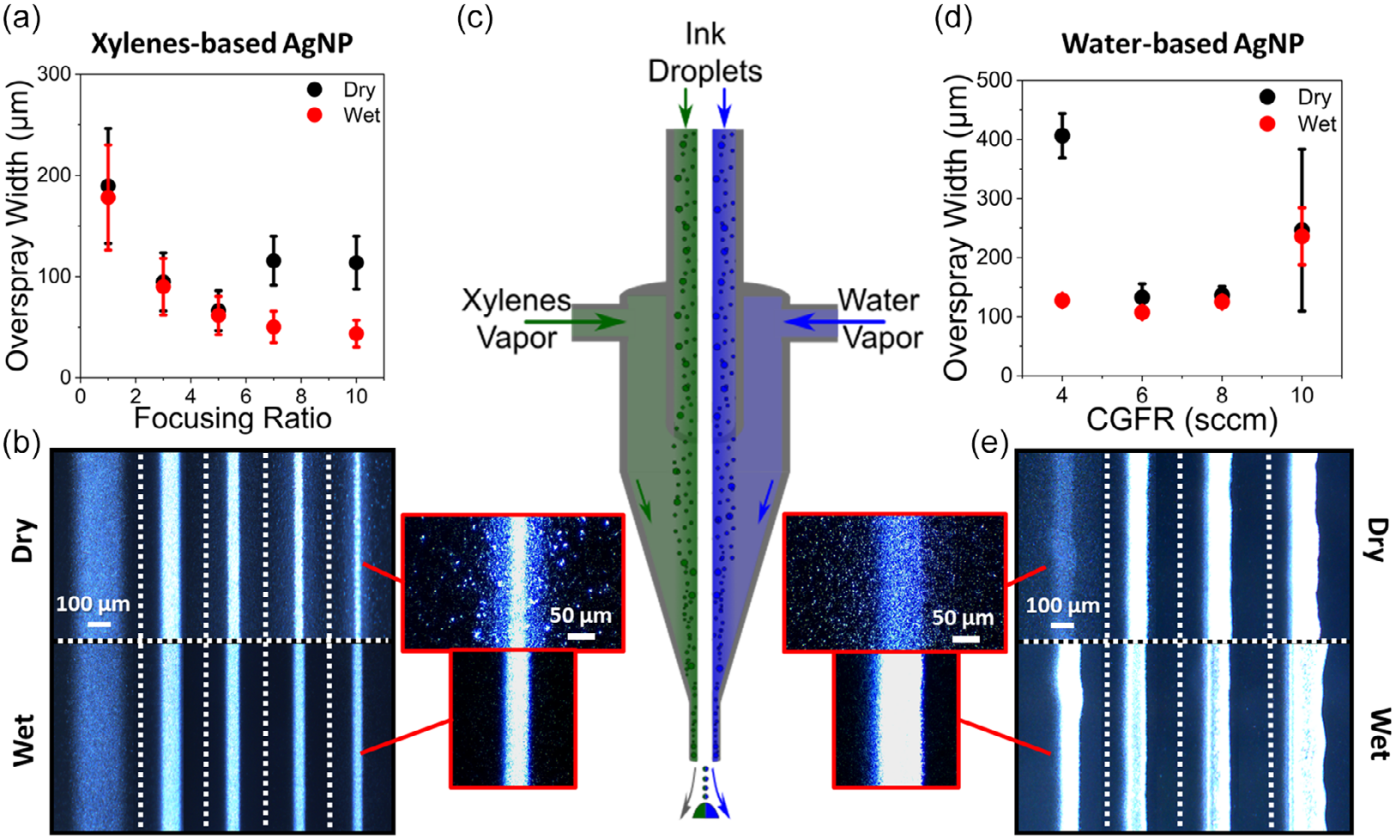
a) UT Dots AgNP ink (xylene‐based) overspray width versus FR (CGFR: 8 sccm, print speed: 2 mm s^−1^, nozzle size: 150 μm). b) Corresponding microscope images. c) Schematic of saturated sheath gas with various solvent chemistries. d) Novacentrix AgNP ink (water‐based) overspray width versus CGFR (FR: 5, print speed: 2 mm s^−1^). e) Corresponding microscope images.

Thus far, the theory of AJP evaporation has been explored with a water‐based polymer ink formulation. To underscore the fundamental nature of this phenomenon in AJP and to broaden the applicability of this methodology, similar experiments were conducted with two distinct silver nanoparticle (AgNP) inks: colloidal dispersions based on xylenes (UT Dots) and an aqueous solvent system containing polymeric dispersants (Novacentrix) (**Figure** **4**). This spans a wide range of ink chemistries and, in the case of water‐based inks in particular, provides a straightforward, cost‐effective pathway toward ink formulation and optimization. In both cases, the bubbler contained solvent corresponding to the primary solvent of the ink: xylenes and deionized water (DIW), respectively. Investigating the print characteristics of these AgNP inks offers a means to benchmark results with the current literature, demonstrate generalization to different ink systems, and validate utility for functional materials more directly applicable to production environments. For the xylenes‐based AgNP ink, overspray was investigated with varying focusing ratios (Figure 4a). Up to a FR of 5, the feature's footprint is relatively similar in both dry and wet conditions; however, above this, the control condition diverged from the predicted trend of reducing line widths as droplets experienced significant drying and were deposited as overspray. By saturating the sheath gas in the most harsh condition (FR = 10) the overspray extent was reduced 62 ± 24%. The trends denoted here correspond well with Figure S2, Supporting Information, depicting droplet drying as a function of focusing ratio when xylenes served as the primary solvent. Here, droplet size is relatively well maintained at the center of the CGF throughout the printhead until nearing a FR of 5. At this point, the saturation ratio falls below the threshold necessary to adequately saturate the gas flow. Discrepancies between the model and experimental results can be attributed to the simulations not accounting for droplet size polydispersity, drying irregularities, and the specific geometry of the actual printhead. The microscope images in Figure 4b further illustrate how the smaller droplets on the outer edge of the carrier gas result in overspray as FR increases. Even though feature size improves marginally, excessive overspray detracts from overall line quality, which is consistent with the outcomes observed for the PI ink at high FRs.

Figure 4d shows the overspray widths measured for the aqueous AgNP ink with varying CGFR. In this case, sheath gas saturation enhanced both line quality and impaction efficiency, as evidenced by the microscope images showing a notable increase in material deposition (Figure 4e). The most significant differences were observed at a relatively low CGFR of 4 sccm, with effects diminishing as a result of increases in the aerosol volume fraction. Higher volume fractions are associated with less drying and spatial variation in droplet size based on numerical modeling (Figure S2, Supporting Information). At higher CGFRs, the printed footprint size increased due to higher deposition rate and liquid phase spreading on the surface. This also resulted in high standard deviations associated with irregularities from line waviness. The outcomes for the Novacentrix ink formulation are less stark than the previous investigations, likely attributed to the presence of a polyurethane binder and cosolvents—glycerol and diethylene glycol—which served to moderate evaporation. Nevertheless, this validates that these improvements derived from sheath gas saturation can be applied to more complex and nonoptimized inks, particularly in conditions where droplet drying is significant.

### 2.4 Relevance of Drying Control for Electronics Fabrication

**Figure 5:**
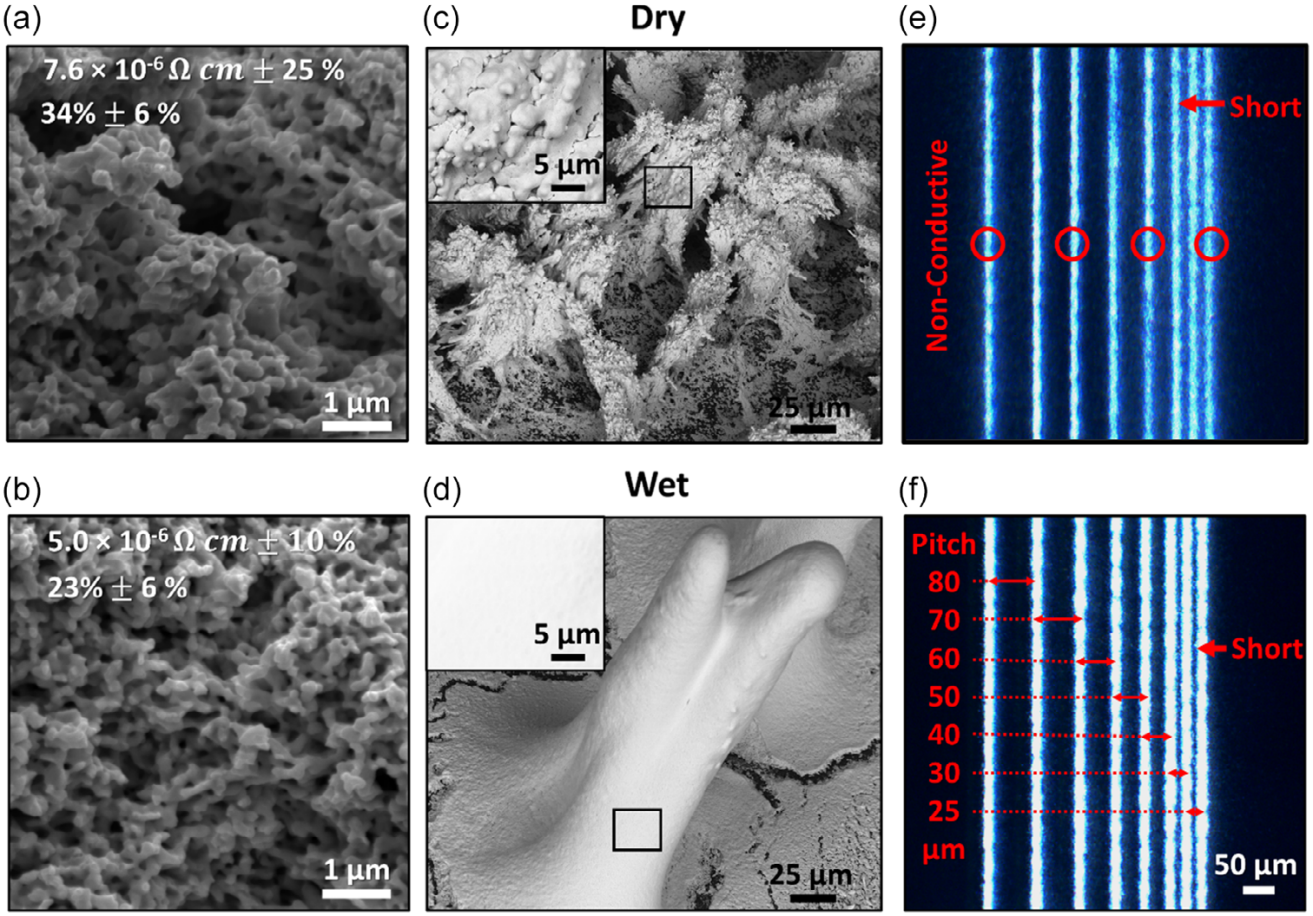
a,b) SEM images of UT Dots AgNP cross section (CGFR: 10 sccm, FR: 5, nozzle size: 200 μm) dry and wet, respectively, annotated with resistivity and porosity values. c,d) SEM images of UT Dots AgNP (CGFR: 10 sccm, FR: 5) dry and wet, respectively. e,f) Microscope images of UT Dots AgNP with 10% terpineol pitch (CGFR: 5 sccm, FR: 12, print speed: 2.5 mm s^−1^, nozzle size: 100 μm) dry and wet, respectively.

The variety of complex, 3D, and conformal electronics applications presents challenges for maintaining high‐quality prints in AJP due to the prevalence of porosity in the microstructure and shorting due to overspray, both of which are exacerbated under dry conditions. With an accompanying theoretical understanding, saturating the sheath gas can provide an additional control for aerosol droplet evolution within the printhead, offering a lever to fine tune these print outcomes. With more solvent in the droplets upon deposition, the fluidity is increased, potentially allowing voids to fill and thus lowering resistivity (**Figure** **5**a,b). This is validated here for a printed AgNP bar, which exhibited a reduction in resistivity of 34 ± 13% when sheath gas saturation was implemented, relative to the control condition. The observed reduction in resistivity can be attributed to the reduction in porosity as analyzed from cross‐sectional SEM images (Figure S3, Supporting Information). The 33% decrease in porosity corresponds well with the reduction of resistivity. This was accompanied by a meaningful reduction in variability between prints, with the relative standard deviation for the resistivity measurement decreasing from 25 to 10% upon implementing sheath gas saturation.

Overspray is particularly prominent in the context of 3D and high aspect ratio geometries that require extended printing in a small region. Maintaining sufficient solvent vapor in the aerosol increases the likelihood that droplets become part of the printed feature, as opposed to being carried away in the gas jet. To demonstrate this, an array of pillars was printed, with each pillar printed completely before moving on to the next (as opposed to a layer‐by‐layer approach building up all pillars concurrently). As shown in Figure 5c,d, SEM images illustrate that printing dry resulted in poorly defined pillars, characterized by overspray and grainy surface structure (inset), whereas the saturated conditions supported fabrication of higher aspect ratios with relatively smooth surface morphology for both the AgNP and PI inks (Figure S4, Supporting Information).

Perhaps the most apparent disadvantage to overspray is the resulting poor line edge quality, which can limit the patterning of closely spaced features. This is relevant across a wide range of high‐demand applications, such as high‐density interconnects. Thus, while resolution is commonly reported as a key metric for AJP, the minimum pitch, or center‐to‐center trace distance, for conductive lines is often a better indicator of print quality. This metric accounts for various factors such as line width variability, overspray, and waviness. To evaluate this, a test pattern (Figure S5, Supporting Information) was developed with varying pitch between parallel lines, and the presence of electrical shorting across adjacent lines was used to evaluate the pitch capabilities. To push toward high‐resolution capabilities, a 100 μm diameter nozzle was used, and the xylenes‐based AgNP ink was modified with 10% v v^−1^ terpineol to assess under more optimal conditions. When printing under standard conditions (i.e., without sheath gas saturation, or “dry”), several of the printed lines were nonconductive owing to localized defects, and electrical shorting was observed for lines printed with a 30 μm pitch. Under the same ink and process parameters but implementing sheath gas saturation (“wet” state), all of the traces were electrically continuous, illustrating better consistency and electrical performance. Moreover, while the core printed traces were empirically wider, the lines were less likely to short due to the reduction in overspray, resulting in an obtainable pitch of 30 μm. This demonstrates the strong value of controlling droplet evaporation to improve functionality for demanding applications in printed electronics, with benefits spanning microstructure, electrical properties, consistency, geometric versatility, and line edge quality. Alongside the aesthetic improvement in feature edge sharpness, and the straightforward benefit of reduced shorting for high‐density interconnects, a reduction in overspray is also posited to benefit resistance to electromigration, performance in high‐frequency electronics, and more general reduction in material contamination on preexisting circuits.^[^ ^11^ ^]^ Overall, sheath gas saturation provides a simple yet effective lever to modulate fundamental mechanisms within the printhead, and when coupled with a theoretical basis to understand and tune its effects, it offers clear potential to better tailor the AJP process.

## 3 Conclusion

This study illustrated the efficacy of presaturating the sheath gas with solvent vapor to mitigate the extent of droplet drying during aerosol jet printing. This significantly diminished overspray extent under dry conditions by nearly half, improving line edge quality and reducing the achievable pitch for high‐density patterns. Reducing the drying extent benefited electrical functionality and microstructural characteristics, reducing resistivity by a third compared to a dry sheath gas. These benefits were observed across a variety of ink chemistries, including both dielectric (water‐based polyimide) and conductive (both water‐based and solvent‐based silver nanoparticle) inks. Saturating the sheath gas shifted the window of operation into previously untenable conditions, facilitating improvements in suboptimal conditions while streamlining the use of unoptimized water‐ and solvent‐based ink formulations. In extremely dry conditions, this approach even offered improvements to well‐established ink formulations by lessening the adverse impacts of smaller, dryer droplets. These experimental results validated our understanding of the influence spatial variation in droplet drying has on print quality and were corroborated by numerical modeling. This combination of theory, computational modeling, and experiments establishes guiding principles and tools to better understand process physics and refine process development for aerosol jet printing. Ultimately, sheath gas saturation lends itself as a straightforward method to modulate evaporation in the aerosol phase, facilitating multifaceted optimization to improve resolution, deposition rate, and morphology for printed and hybrid electronics fabrication.

## 4 Experimental Section

4.1

4.1.1

##### Materials

A polyimide stock solution was purchased from UT Dots (UTD‐PI‐SD) and diluted with deionized water (DIW) in a 0.72:2.28 v v^−1^ ratio (stock:DIW) to prepare the ink. An aqueous silver nanoparticle ink containing a polyurethane binder was purchased from Novacentrix (JS‐A426) and diluted with DIW in a 2:8 v v^−1^ ratio (stock:DIW). A xylenes‐based silver nanoparticle dispersion was purchased from UT Dots (UTD‐Ag25X) and mixed with xylenes in a 1:4 v v^−1^ ratio (stock:xylenes) for most experiments. For the high‐resolution pitch test (Figure 5e–f), terpineol was included in the ink, in a 2:7:1 v v^−1^ ratio (stock:xylenes:terpineol). Xylenes and terpineol were purchased from Millipore Sigma. 200 proof ethyl alcohol was sourced from Fisher (# 04‐355‐223).

##### Sample Preparation

All prints were fabricated with a custom 3‐axis printer and ultrasonic atomizer. A light scattering sensor was implemented in line with the printhead to reduce process drift and improve reliability of printed outcomes.^[^ ^35^ ^]^ Two Alicat mass flow controllers modulated the flow of nitrogen for the carrier gas and sheath gas flows. A bubbler housing solvent analogous to the ink chemistry (DIW for PI and aqueous AgNP, and xylenes for AgNP) was placed along the sheath gas line. In the bubbler, a porous filter stone was used to introduce gas bubbles through the liquid to partially saturate the sheath gas with solvent vapor upstream of the flow cell. The bubbler was held at 25 °C for water and 20 °C for xylenes. The ink cartridge was made of a photopolymer SLA resin (clear resin, Formlabs) which sat within an atomizer bath at 20 °C. Unless otherwise noted, all samples were printed with a 250 μm nozzle onto glass slides, which were cleaned with ethyl alcohol and held on a 60 °C print bed. Following printing, PI samples were cured on a hotplate at 100, 150, and 200 °C sequentially in 30 min segments, while the AgNP samples were sintered at 250 °C for 1 h on a hotplate in air. A table of printing conditions associated with each figure in the manuscript can be found in the Supporting Information (Table S1).

##### Characterization

General characterization and porosity tests with the PI and AgNP inks had a sample size of *n* = 3, while the pillar and pitch experiments had a sample size of *n* = 1. Error bars indicate standard deviation between the printed samples. Overspray width was calculated across a 0.84 mm line length using a MATLAB code. This length represents the middle 60% of an image taken at 5× magnification to eliminate variation in lighting. In this process, images collected in dark field with a Motic optical microscope were converted to a binary based on a common threshold value to identify the overspray of samples printed at 1, 2, and 5 mm s^−1^. This was verified by an operator, and in the limited case of lower deposition rates, the threshold level was occasionally adjusted to accurately distinguish overspray. Overspray width denotes the spatial difference between the 2nd percentile and 98th percentile of the white pixels to reduce the effects of background irregularities (Figure S6, Supporting Information). Cross‐sectional areas were acquired using an optical profilometer (Zygo NewView 9000) and analyzed using a python script. Electrical measurements for conductivity calculation were taken using a four‐point probe configuration with a Keithley 2450 source meter, with the sample comprised of 10 overlapping traces to reduce the experimental uncertainty for cross‐sectional area.

## Conflict of Interest

The authors declare no conflict of interest.

## Author Contributions

**Bella I. Guyll**: formal analysis (lead); funding acquisition (supporting); investigation (lead); methodology (equal); supervision (supporting); visualization (lead); writing—original draft (lead). **Brayden L. Sanford**: formal analysis (supporting); investigation (supporting). **Cary L. Pint**: supervision (supporting); writing—review and editing (supporting). **Ethan B. Secor**: conceptualization (lead); funding acquisition (lead); methodology (equal); resources (lead); supervision (lead); writing—review and editing (lead).

## Supporting information

Supplementary Material

## Data Availability

The data that support the findings of this study are available from the corresponding author upon reasonable request.
