# Supplementary material for: Controlling Droplet Evaporation in Aerosol Jet Printing to Understand and Mitigate Overspray
Source: Small Sci. 2025 Mar 18;5(7):2500069. doi: 10.1002/smsc.202500069 (PMC12257901; doi:10.1002/smsc.202500069)
Supplement: Supplementary file 1 — Supplementary Material [file SMSC-5-2500069-s001.pdf]

Supporting Information

Controlling Droplet Evaporation in Aerosol Jet Printing to Understand and Mitigate Overspray

*Bella I. Guyll, Brayden L. Sanford, Cary L. Pint, Ethan B. Secor**

B.I. Guyll, B.L. Sanford, C.L. Pint, E.B. Secor
Department of Mechanical Engineering
Iowa State University
Ames, Iowa 50011, USA
Email: esecor@iastate.edu

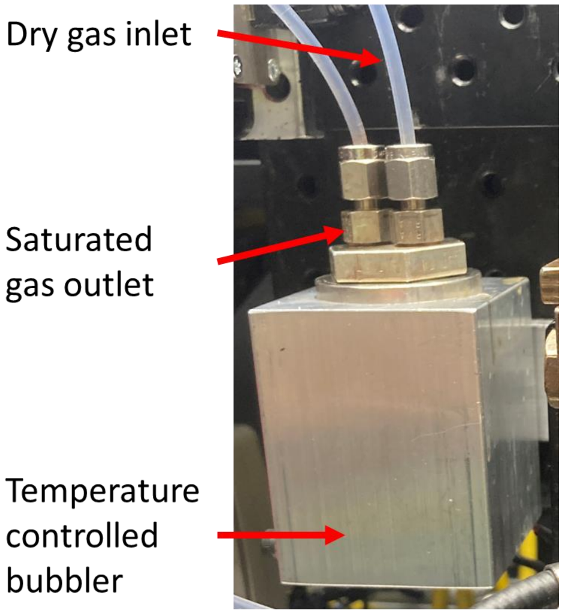

Figure S1: Bubbler set up used for experiments.

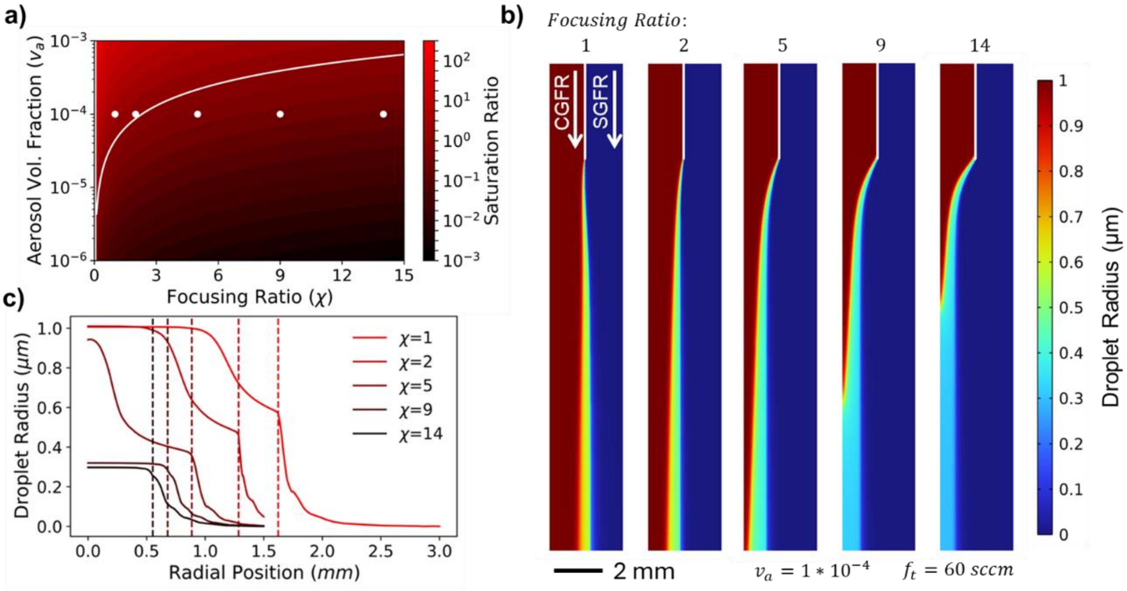

Figure S2: Theoretical analysis of droplet evaporation. (a) Saturation ratio plotted as a function of aerosol volume fraction and focusing ratio for xylenes, showing the driving force for bulk evaporation. (b) Plots of the droplet size as a function of position for numerical simulations. The interior carrier gas flow (CGF) contains droplets, and after joining the annular sheath gas flow (SGF) these droplets begin evaporating. Simulation results are shown for five conditions with different focusing ratios (shown in (a)), but equivalent total flow rate and aerosol volume fraction. (c) Droplet size at the chamber exit, plotted against radial position. The dashed line indicates the edge of the droplet flow, with nonzero values outside this region arising from numerical diffusion.

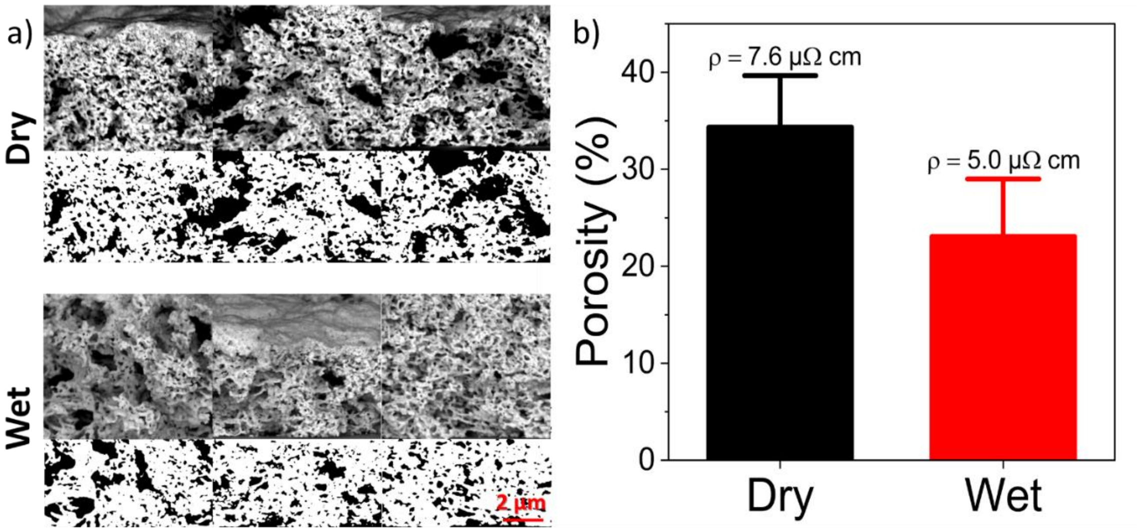

Figure S3: (a) SEM cross-section images of 10-pass printed AgNP traces (CGFR: 10 sccm, FR: 5, print speed: 2.5 mm s^{-1}, nozzle size: 200 µm) and corresponding ImageJ binary analysis in dry (top) and wet (bottom) conditions. (b) Porosity measured from the AgNP cross-section and the samples' corresponding resistivity values.

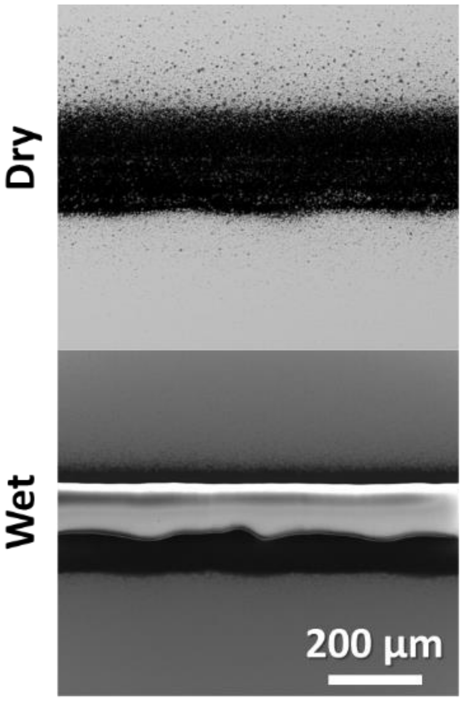

Figure S4: SEM top-view images of printed PI ink on a silicon wafer (CGFR: 7 sccm, FR: 10, print speed: 2.5 mm s^{-1}, nozzle size: 200 µm) in dry (top) and wet (bottom) conditions.

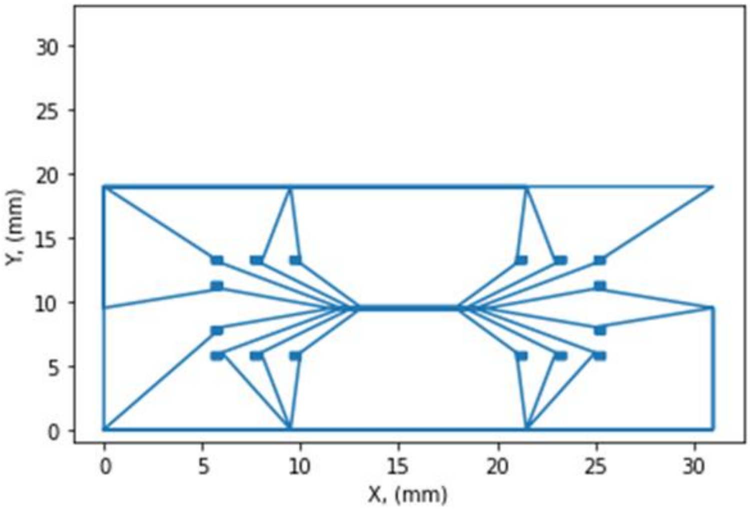

Figure S5: Toolpath for pitch experiments. Connecting lines around the perimeter are masked off with tape during printing. Conductivity is measured for each line, and conductance between adjacent lines (shorting) is tested to determine the minimum achievable pitch value.

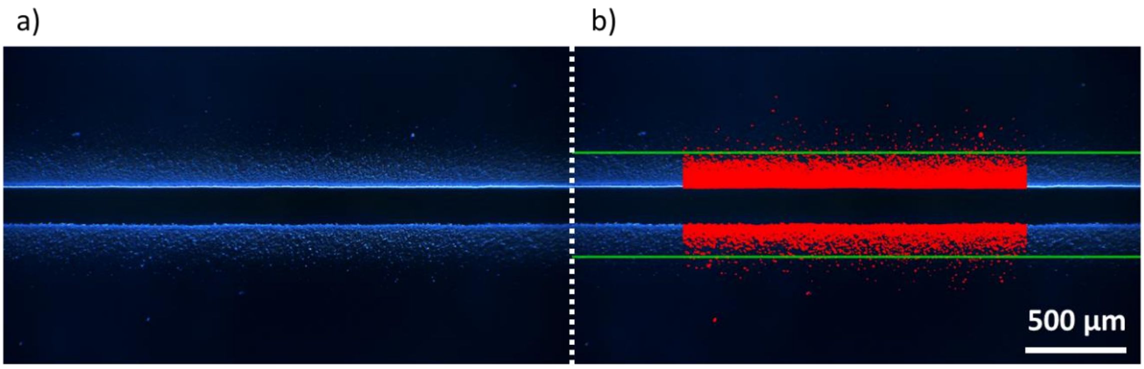

Figure S6: Overspray width analysis. (a) Original dark-field microscope image. (b) Analyzed image with red indicating pixels deemed overspray within the center 60% of the printed trace. Horizontal green lines represent the spatial location of the 2nd and 98th percentile of droplets. The distance between the two lines is the quantitative metric described herein as the "overspray width."

Table S1. Printing conditions associated with each figure.

Figure	Material	Sample Number	CGFR(s)	FR(s)	Speed(s)	Nozzle Size
Figure 1b	PI - water	3	6, 8, 10, 12, 14	5	2 mm s^{-1}	200 µm
Figure 1c	PI - water	3	6, 8, 10, 12, 14	5	2 mm s^{-1}	200 µm
Figure 2a	PI - water	3	10	3, 5, 7	2 mm s^{-1}	200 µm
Figure 2b	PI - water	3	10	1, 3, 5, 7, 10	1, 2, 5 mm s^{-1}	200 µm
Figure 2c	PI - water	3	10	3, 5, 10	1, 2, 5 mm s^{-1}	150 µm
Figure 4a,b	AgNP - xylenes	1	8	1, 3, 5, 7, 10	2 mm s^{-1}	150 µm
Figure 4d,e	AgNP - water	3	2, 4, 6, 8, 10	10	2 mm s^{-1}	200 µm
Figure 5a,b	AgNP - xylenes	3	10	5	2.5 mm s^{-1}	200 µm
Figure c,d	AgNP - xylenes	2	10	5		250 µm
Figure e,f	AgNP (2:7:1) xylenes	1	5	12	2.5 mm s^{-1}	100 µm
